# Trade-offs between performance and variability in the escape responses of bluegill sunfish (*Lepomis macrochirus*)

**DOI:** 10.1242/bio.201511577

**Published:** 2015-04-24

**Authors:** Amanda C. Hitchcock, Tiffany Chen, Erin Connolly, Karin Darakananda, Janet Jeong, Arbor Quist, Allison Robbins, David J. Ellerby

**Affiliations:** Department of Biological Sciences, Wellesley College, 106 Central Street, Wellesley, MA 02481, USA

**Keywords:** Fish, Escape, Behaviour, Biomechanics, Trade-offs, *Lepomis macrochirus*

## Abstract

Successful predator evasion is essential to the fitness of many animals. Variation in escape behaviour may be adaptive as it reduces predictability, enhancing escape success. High escape velocities and accelerations also increase escape success, but biomechanical factors likely constrain the behavioural range over which performance can be maximized. There may therefore be a trade-off between variation and performance during escape responses. We have used bluegill sunfish (*Lepomis macrochirus*) escape responses to examine this potential trade-off, determining the full repertoire of escape behaviour for individual bluegill sunfish and linking this to performance as indicated by escape velocity and acceleration. Fish escapes involve an initial C-bend of the body axis, followed by variable steering movements. These generate thrust and establish the escape direction. Directional changes during the initial C-bend were less variable than the final escape angle, and the most frequent directions were associated with high escape velocity. Significant inter-individual differences in escape angles magnified the overall variation, maintaining unpredictability from a predator perspective. Steering in the latter stages of the escape to establish the final escape trajectory also affected performance, with turns away from the stimulus associated with reduced velocity. This suggests that modulation of escape behaviour by steering may also have an associated performance cost. This has important implications for understanding the scope and control of intra- and inter-individual variation in escape behaviour and the associated costs and benefits.

## INTRODUCTION

Effective predator evasion is a vital component of fitness for many animals (Husak, 2006; Miles, 2004; Walker et al., 2005; Watkins, 1996). Given the high cost of failure, intense selection pressures are expected to favour biomechanical traits and escape strategies that increase the likelihood of escape (Domenici et al., 2011a; Lind and Cresswell, 2005; Weihs and Webb, 1984). The physical and physiological features that drive escape responses may be optimized for creating high power outputs and accelerations (Aerts, 1998; Askew and Marsh, 2002; Henry et al., 2005; Nauen and Shadwick, 2001; Roberts and Marsh, 2003; Sutton and Burrows, 2011), performance traits associated with escape success (Husak, 2006; Walker et al., 2005). Mechanical performance is not the only predictor of escape success: theoretical models have identified optimal strategies, particularly in terms of the escape direction relative to a predator (Arnott et al., 1999; Domenici, 2002; Weihs and Webb, 1984); escape behaviour may be modulated in response to changing environmental factors (Domenici, 2010a); and variation in behaviour may be important to avoid predictability (Domenici et al., 2008). Although mechanical performance, behavioural variation and the scope for behavioural modulation all affect escape success and organismal fitness, the interaction of these factors is poorly understood (Wainwright et al., 2008).

Escape performance is dictated by a suite of interacting physical factors. For example, during fish escape responses rapid muscle contraction and bending of the body axis transfers momentum to the surrounding water, potentially generating high escape velocities (Webb, 1978; Domenici and Blake, 1991; Domenici and Blake, 1993). In complex, coupled systems of this type, the scope for behavioural variation can be limited (Wainwright et al., 2008). Axial kinematics are constrained, as the mechanical properties of the axial skeleton and associated connective tissue limit the extent of body curvature (Nowroozi and Brainerd, 2013; Westneat et al., 1998), and the contractile properties of the myotomal musculature and inertia of the tissues and surrounding water determine the rate at which the body axis can bend (Wakeling and Johnston, 1999). Flow patterns associated with thrust production are initiated by the first body bend and continue to develop through subsequent kinematic stages of the escape. Steering during these latter stages to modulate the escape trajectory can limit power transfer to the water (Tytell and Lauder, 2008). The pattern of possible body movements is therefore constrained, as are the mechanisms for translating them into thrust. This may create a trade-off between variation in escape behaviour and mechanical performance, where high performance is limited to a relatively narrow range of kinematic variation, and modulation of behaviour to increase variability may have an associated performance cost.

Despite the potential physical constraints, fish escape responses appear to be quite variable (Wöhl and Schuster, 2007; Domenici, 2010a; Domenici et al., 2011a; Domenici et al., 2011b; Marras et al., 2011). This may be adaptive, as stereotyped escape responses allow predators to anticipate prey behaviour (Jabłoński and Strausfeld, 2001). Individual behavioural repertoires are difficult to assess, however, as most datasets are composites obtained by pooling relatively small numbers of observations across groups of individuals (Domenici and Batty, 1997; Domenici and Blake, 1993; Eaton and Emberley, 1991; Eaton et al., 1988; Foreman and Eaton, 1993; Gerry et al., 2012; Kasapi et al., 1993; Meager et al., 2006; Walker et al., 2005), and do not indicate if the overall scope of behavioural variation is due to similarly variable patterns of behaviour across individuals, or pooled differences between individuals. From an evolutionary perspective it is important to be able to quantify and distinguish between levels of inter- and intra-individual variation. Selection on escape behaviour, assuming a link between escape performance and fitness, requires both inter-individual variation and relative consistency of behaviour within individuals. Assessing repeatability, an indicator of the extent to which variation within individuals contributes to total variation in the population and an indicator of the upper level of heritability for a behavioural phenotype (Lessels and Boag, 1987), would allow variation in escape behaviour to be placed in context with other types of vertebrate behaviour, and indicate the extent to which escape behaviour can be shaped by selection.

The goals of the current study were to quantify the extent of individual variability in fish escape behaviour, place this in context with the overall scope for behavioural variability across individuals, and determine the extent to which behavioural variation and flexibility were constrained by a trade-off between variability and mechanical performance. Data to indicate the overall scope for intra- and inter-individual variation in escape behaviour and allow determination of behavioural repeatability for fish escapes are scarce (Domenici 2010b), and based on either small numbers of observations, or single, best-performance observations compared between time points (Gibson and Johnston, 1995; Marras et al., 2011), which may underestimate the overall scope for variation. To our knowledge, there are no data that place escape performance data in context with an individual's scope for variation in escape behaviour.

In the present study we have quantified the repertoire of escape behaviours for bluegill sunfish (*Lepomis macrochirus*), encompassing the intra- and inter-individual variation in escape angle, and linked this to escape performance, specifically velocity and acceleration, variables associated with both escape success (Walker et al., 2005) and effective power transfer to the water (Webb, 1978; Tytell and Lauder, 2008). High-speed video analysis of multiple escape responses for each individual allowed the distribution of escape angles to be determined and compared between individuals, and concurrent analyses of centre of mass displacement enabled a comparison between mechanical performance and the probability of the associated kinematics within the observed frequency distribution of escape movements. This enabled us to test the following hypotheses: first, that the variation of escape behaviour in individuals is relatively constrained and that inter-individual variation increases the apparent overall scope for variability; and second, that there is a performance cost associated with variability in escape behaviour. This cost may be manifested in two ways: first, through the restriction of high velocities and accelerations to a relatively narrow range of kinematic variables, and second through a reduction in performance associated with steering behaviour that increases variation in the final escape direction. These analyses have important implications for assessing the relative costs and benefits of variable escape behaviour, where the variation required for unpredictability and behavioural flexibility to seek refuge or maximize distance from a predator may be incompatible with high performance.

## RESULTS

**Fig. 1.**
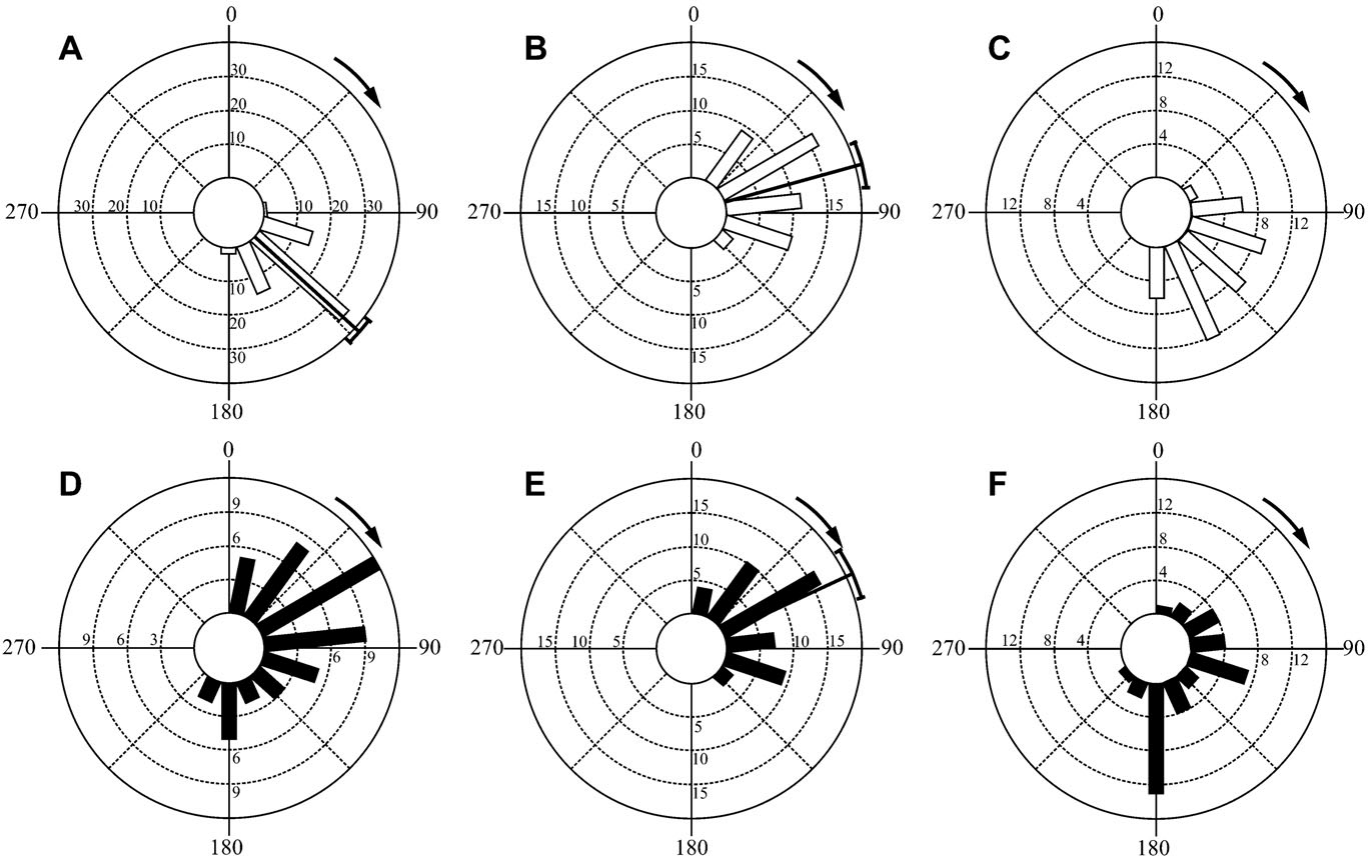
Inter-individual variation in the escape angle frequency distributions of bluegill sunfish. Radial axes show the number of observations within 24° bins. Data are shown for stage 1 angles (open bars, A, B and C) and final escape angles (black bars, D, E and F) from three representative fish. For these individuals no significant differences in angle distribution were detected for left and right turns and both were combined into a single distribution. Panels are paired A and D, n = 51, B and E, n = 68, and C and F, n = 40. The mean±95% confidence interval is shown where the distribution was not detectably different from a circular normal distribution. The stimuli were delivered at 0°, directly in front of and in line with the long axis of the fish. Arrows indicate the turn direction from 0°.

**Fig. 2.**
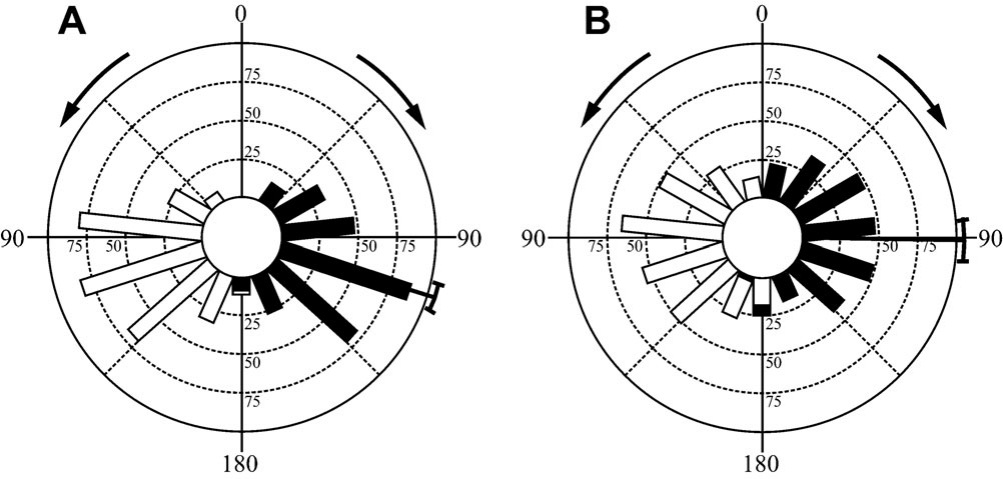
Composite circular frequency distributions for (A) stage 1 angles and (B) final escape angles of bluegill sunfish. Radial axes show the number of observations within 24° bins. Right and left turns are shown by black and open bars respectively. Data are from 14 fish, total number of escape responses = 604. The mean±95% confidence interval is shown where the distribution was not detectably different from a circular normal distribution. The stimuli were delivered at 0°, directly in front of and in line with the long axis of the fish. Left and right arrows indicate the turn direction from 0° for the white and black distributions respectively.

Inter-individual variation in behaviour (Fig. 1) magnified the variation in the overall sample of escape responses pooled across individuals (Fig. 2). For the combined stage 1 angles circular variance was 0.16, significantly greater than the sample of circular variances obtained for individual fish (mean 0.12, range 0.04 to 0.21; one-sample t-test, *t*(14) = 2.49, p<0.05). For the composite distribution of final angles the circular variance of 0.30 was also significantly greater than in individual distributions (mean 0.23, range 0.07 to 0.39; one-sample t-test, *t*(14) = 2.71, p<0.05, Figs 1, 2). This pattern of inter-individual variation is further supported by pair-wise comparisons of stage 1 and final escape angles, where of the 105 pair-wise comparisons possible with 15 individuals, 71 detected significant differences (Mardia-Watson-Wheeler, p<0.02, p adjusted with Ryan's Q; Figs 1, 2). Significant inter-individual differences in escape angles were also indicated by ANOVA (Table 1). Non-zero repeatability values were calculated for all directional and COM motion variables with the exception of peak COM acceleration (Table 1). These ranged from 0.09 for steering angle, to a maximum of 0.43 for displacement.

**Table 1.**
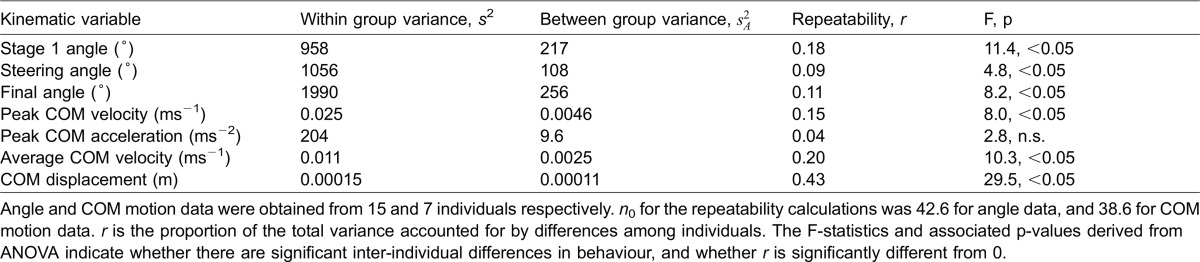
Repeatability estimates for angular changes and COM motion parameters during escape responses

**Fig. 3.**
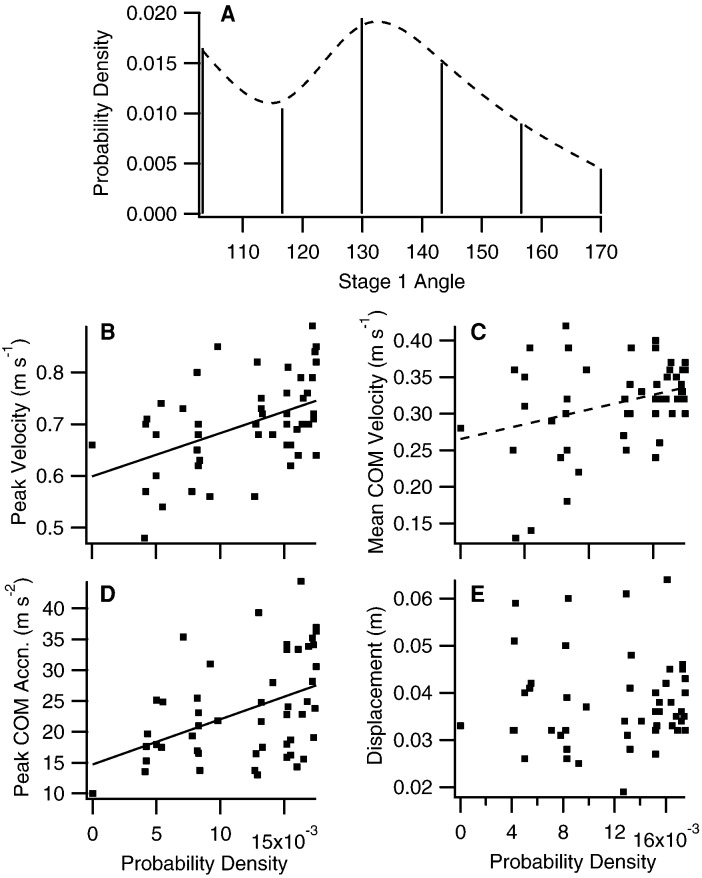
Relationships between performance and the frequency distribution of stage 1 escape response angles in bluegill sunfish. (A) Histogram of a representative stage 1 angle frequency distribution. Frequencies are normalized to a probability density with an integral of 1. The frequency distribution was fitted with a fourth order polynomial (dashed line) to estimate the probability density function of the stage 1 angles. Relationships between (B) peak COM velocity, (C) mean COM velocity, (D) peak COM acceleration, and (E) COM displacement during escape responses and the probability density for the corresponding stage 1 angles. Unbroken lines denote a positive PPMC significant at the 0.05 level and a slope of the linear relationship detectably different from 0. The dashed line represents a positive PPMC significant at the 0.05 level, and a slope not detectable different from 0. Data are for a single representative fish. n = 50.

Escape performance was associated with the frequency with which stage 1 escape angles were used by an individual (Fig. 3). Peak COM velocity was significantly and positively correlated with the probability density of the stage 1 angle frequency distributions in all individuals (Table 2, Pearson product-moment correlation, PPMC, p<0.05), and the slopes derived from the linear regression analyses were detectably different from zero (Table 2, t-test, two-tailed p<0.05). Treating the PPMCs as a measure of effect size (Cohen, 1988), their sign and magnitude suggested a positive association between both peak COM acceleration and average COM velocity and the stage 1 probability density functions. This was less apparent than for peak COM velocity, and the PMCC was not statistically significant at the α = 0.05 level, and/or the slope of the linear relationship was not detectably different from zero in a subset of the individuals (Table 2). There was no detectable relationship between COM displacement and the stage 1 probability density function. There were also no detectable relationships between performance and the probability density functions describing the frequency distributions of the final escape angle (data not shown).

**Table 2.**
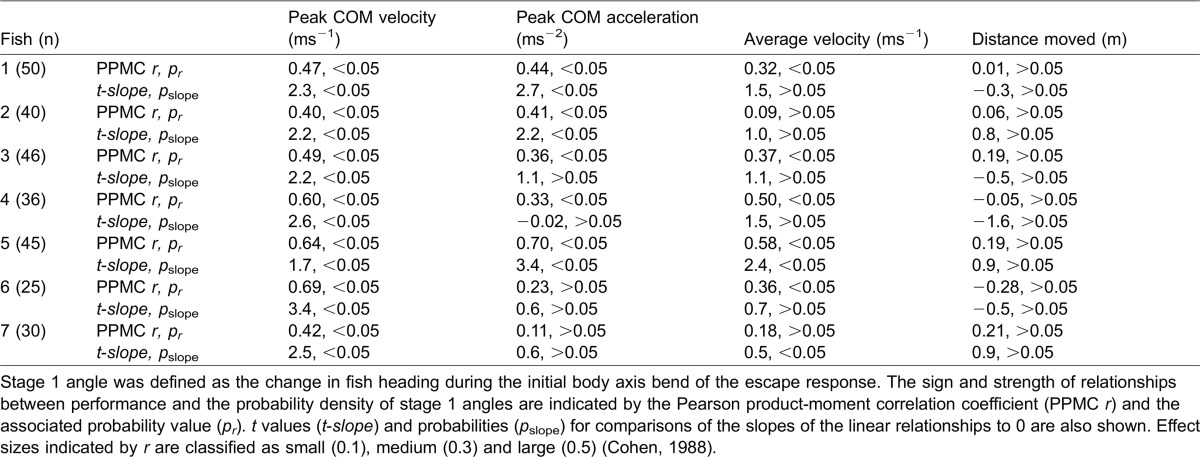
Relationships between escape performance and the probability density of stage 1 angle distributions

**Fig. 4.**
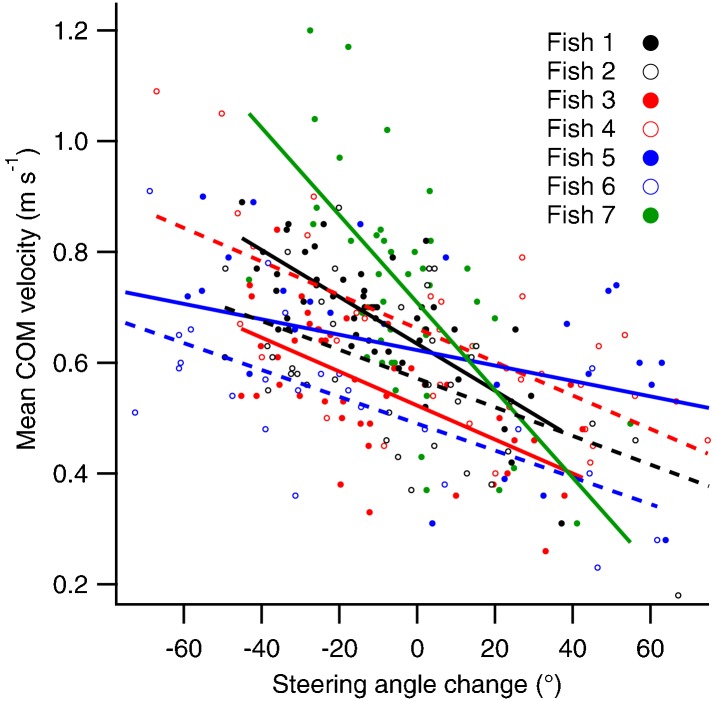
Relationships between the mean centre of mass velocity and steering angle during escape responses in bluegill sunfish. The steering angle was the change in fish heading between completion of the initial stage 1 C-bend and establishment of the final escape trajectory. Positive angles represent a continuation of the initial turn direction, and negative angles a reversal in turn direction. Linear relationships are fitted to the data from individual fish. Unbroken lines are associated with closed symbols, and broken lines with open symbols of the same colour.

Circular variance was significantly greater for final compared to stage 1 distributions (paired t-test, t(14) = 5.98, p<0.05; Figs 1, 2), suggesting an increase in variation imposed by steering subsequent to the initial stage 1 C-bend. The magnitude and direction of steering after the stage 1 C-bend was also associated with changes in performance (Fig. 4). Average COM velocity decreased significantly with steering angle (Table 3, PPMC, p<0.05), and the slopes derived from the linear regression analyses were detectably different from zero with one exception (Table 3, t-test, two-tailed p<0.05). A negative relationship between peak COM velocity and steering angle was also suggested by the magnitude of the PPMCs as a measure of effect size (Cohen, 1988), although this was not consistently confirmed at the α = 0.05 level. Peak COM acceleration and distance moved by the COM showed no detectable relationship to steering angle (Table 3).

**Table 3.**
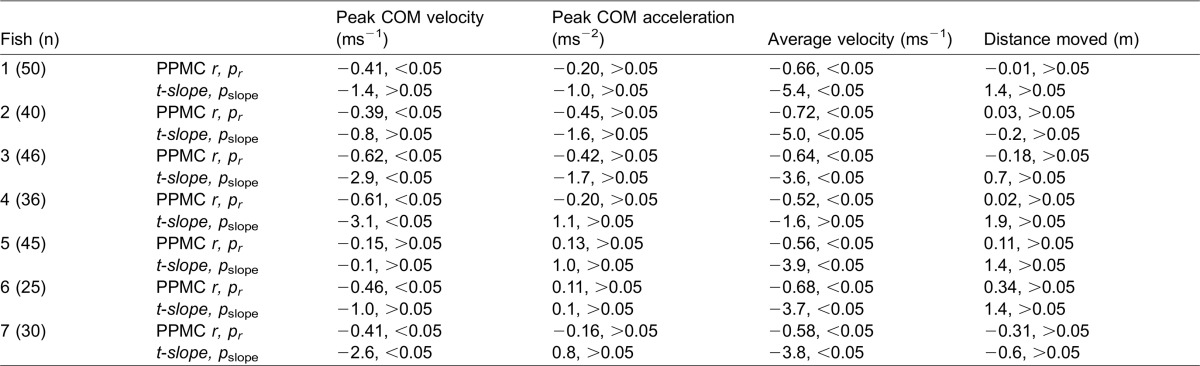
Relationships between escape performance and steering to change the fish heading subsequent to stage 1

## DISCUSSION

Fish most frequently use escape angles that are associated with high performance. This suggests that a high level of variation in escape behaviour is associated with a performance cost (Fig. 3; Table 2). Performance constraints are likely imposed by the central role of stage 1 kinematics in the effective transfer of momentum to the water. Although this stage has been referred to as ‘preparatory’, and viewed largely as a turning manoeuvre to control escape angle, and/or a pre-positioning of the body axis for maximum thrust generation by the tail during subsequent countermovement of the body axis (Eaton and Emberley, 1991; Eaton et al., 1988; Weihs, 1973), hydrodynamic analyses show that much of the momentum transfer to the water associated with accelerating the body occurs during stage 1 (Tytell and Lauder, 2008; Borazjani et al., 2012). The nature of the flow patterns generated during stage 1 and their continued development during stage 2 means that there may be limited scope for variation in the kinematics of stage 1 and the timing of progression to stage 2 without compromising thrust generation. This likely explains why stage 1 kinematics are relatively constrained, with some angles never being employed (Figs 1, 2), and the less frequently used angles being associated with lower escape velocities (Table 2; Fig. 3).

Variation in the later stages of the escape response may also affect performance, although the relationship to direction is different to that for stage 1. Final escape angles achieved by a net increase in angle after stage 1 are associated with lower performance than where the initial turning direction is reversed (Table 3; Fig. 4). A similar pattern was detected in angelfish (*Pterophyllum eimekei*) where ‘single bend’ escapes that lack a stage 2 counter-turn were associated with lower peak velocities than ‘double bend’ escapes where stage 2 was present (Domenici and Blake, 1991). Continued turning in the initial direction is achieved in two ways. First, by a weakly defined stage 2, impairing thrust production (Tytell et al., 2008) and the further increase in velocity associated with stage 2 (Domenici and Blake, 1991). Second, through the addition of a further turn away from the stimulus after stage 2, prolonging the time taken to attain a final escape direction and restricting the average velocity (Fig. 4). Accelerations typically peak during stage 1 (Domenici and Blake, 1991), and are therefore less tightly coupled to subsequent steering behaviour than velocity (Table 2).

If maximum performance is limited to a relatively narrow kinematic range, and high velocities and accelerations are associated with escape success (Walker et al., 2005), why do fish sometimes employ less effective kinematics? Variation and unpredictability are also important aspects of escape success as predators can potentially exploit stereotyped prey behaviours (Catania, 2009; Jabłoński and Strausfeld, 2001). Unpredictability would be maximized by random escape angles (Humphries and Driver, 1970). Given the constraints on the system randomness is clearly not achievable. The limits to variation at the individual level are in part alleviated by significant inter-individual differences in escape kinematics (Fig. 1; Table 1). These differences result in an overall, composite pattern of variation that is greater than that shown by most individuals (Fig. 2). The combination of inter- and intra- individual variation in escape behaviour may therefore be adaptive in creating unpredictability from a predator perspective despite limits to behavioural variation imposed by proximate, mechanical constraints on performance.

Flexibility, the ability to modulate behaviour in response to changing conditions, may also be significant in certain circumstances (Wainwright et al., 2008). Much of the flexibility in the escape response resides in kinematic events after stage 1, indicated by the increased variance and reduced repeatability of final escape angles in comparison to stage 1 angle (Figs 1, 2; Table 1). Although mechanical performance can predict escape success (Walker et al., 2005), movement relative to a predator is also important (Weihs and Webb, 1984). As the stimulus was delivered directly in front of the fish, all stage 1 turns are away from the ‘threat’. Although a greater turn away from the stimulus results in lower performance, it would maximize the predator-prey distance, while a thrust enhancing, stage 2, counter-turn moves the heading of the fish back towards the initial stimulus. This suggests a trade-off between high performance and steering imposed by the requirement for a stage 2 counter turn to enable further development of thrust-associated flow patterns initiated during stage 1 (Borazjani et al., 2012). Escape behaviour may be modulated not only in response to predator trajectory, but also with regard to the presence of shelter (Zani et al., 2009) or conspecifics (Hall et al., 1986), or to maintain sensory contact with the predator (Domenici and Blake, 1993). Escapes modulated to account for these factors may suffer impaired performance if they require the fish to adopt an escape direction or steering manoeuvres that are incompatible with effective thrust generation and maintenance of high velocities. Flexibility, in addition to variability, may therefore incur a performance cost.

Repeatability (*r*) indicates the proportion of phenotypic variability attributable to differences between individuals. It also sets the upper limit for the heritability of a given trait (Lessells and Boag, 1987). High *r* values result from consistency of behaviour for a given individual and/or relatively large inter-individual differences in behaviour with a value of 1 indicating different individual behaviours that are perfectly consistent. Conversely, low *r* values indicate low individual consistency and/or relatively small differences between individuals, 0 indicating no inter-individual difference in behaviour. Bluegill escape repeatabilities fall at the lower end of the range of repeatabilities or equivalent intraclass correlation coefficients reported for escape performance in other species. For example, in the western mosquitofish (*Gambusia affinis*; Langerhans et al., 2004) burst speed repeatability was 0.89, 0.22 to 0.44 in red drum larvae depending on stimulus type (*Sciaenops ocellatus*; Fuiman and Cowan, 2003), and in sprinting lizards intraclass correlations range from 0.24 to 0.97 (Garland, 1985; Gleeson and Harrison, 1988; Bonine and Garland, 1999). This may in part be a taxonomic association. Repeatabilities for a wide range of behaviours are lower on average in fish than in amphibians and amniotes (Bell et al., 2009). Methodological differences are also a likely factor. The present study was based on large numbers of observations per individual, whereas reported escape performance repeatabilities are typically based on a small number of observations, or the best measured performance at a given time point. Larger numbers of observations are likely to increase the measured scope for individual variation in behaviour, particularly for fish escapes as these are intrinsically variable, particularly with regard to escape direction (Domenici and Blake, 1991; Domenici and Blake, 1997), with an associated reduction in repeatability. A final factor may be a relatively small difference in inter-individual performance levels. Despite non-zero repeatabilities and significant inter-individual differences for most performance variables (Table 1), the range of mean performance values was relatively narrow (e.g. 0.54 to 0.73 ms^−1^ peak velocity, 20.4 to 29.7 ms^−2^ peak acceleration) with an absence of consistently poor performers. As these were wild-caught fish, this may reflect narrowing of the scope for inter-individual variation through removal of low performance phenotypes (Fuiman and Cowan, 2003).

Given the potential many-to-many mapping of physical and physiological features to various aspects of escape performance intra- and inter-individual variation in escape behaviour may arise from a combination of factors. The stage 1 C-bend is controlled by paired reticulospinal Mauthner neurons and associated command neurons (Eaton et al., 2001; Korn and Faber, 2005). Outputs from the Mauthner neurons themselves may be stereotyped (Nissanov et al., 1990), but activity in associated segmental homologs of the Mauthner cells in the hind brain, and an associated network of descending neurons (Gahtan et al., 2002; Metcalfe et al., 1986; O'Malley et al., 1996; Weiss et al., 2006) is variable and may control escape kinematics and direction. Stage 1 angle correlates with the duration and intensity of electrical activity in the myotomal muscle producing the initial C-bend (Eaton et al., 1988), so modulation of activity in the neural networks controlling muscle contraction could impose both intra- and inter-individual variability. Further inter-individual variation may be created by differences in muscle mass and contractile properties, and the mechanical properties of connective tissues, which dictate the form of the C-bend (Webb, 1978). During the C-bend both the body and fins contribute to momentum transfer to the water (Tytell and Lauder, 2008; Tytell et al., 2008). Bluegill sunfish show variation in body and fin shape within populations (Gerry et al., 2011) that are associated with differences in escape performance (Gerry et al., 2012), and this may further contribute to differences in the relationship between body kinematics and thrust generation.

### 

#### Conclusions

Escape responses are both varied and flexible, and both variation and flexibility have a performance cost. The most frequently used kinematics during the initial, C-bend of the bluegill escape response are associated with the highest escape velocities. This creates a trade-off between kinematic variation and mechanical performance. The predictability of relatively constrained escape movements could reduce escape success, but this is alleviated by the increased overall scope for variability created by inter-individual variation in behaviour. Further variation is imposed by steering in the latter stages of the escape. This may also be associated with a trade-off where steering to increase distance from the initial stimulus reduces the overall escape velocity.

## MATERIALS AND METHODS

Bluegill sunfish (*Lepomis macrochirus* Rafinesque) were collected from Lake Waban, MA, USA using baited hooks in August and September 2011. Fish were maintained in pairs in divided 20 gallon aquaria at 21°C, and fed on earthworms *ad libitum*. Kinematic data indicating changes in fish heading through the escape response were obtained from 15 fish (body mass 104±13 g, mean±s.d.) with sufficient numbers of observations per individual to establish the frequency distribution of escape angles. Analyses of velocities and accelerations were carried out for 7 of these fish (body mass 101±8 g, mean±s.d.). Fish were collected under license from the Massachusetts Department of Fish and Game, and all procedures were approved by the Institutional Animal Care and Use Committee at Wellesley College.

### Kinematic analyses

Video recordings were obtained in a 45×90 cm tank with 15 cm water depth (Gerry et al., 2012). Escapes were recorded from above using an AOS X-PRI camera (AOS Technologies, Baden Daettwil, Switzerland) at a frame rate of 500 Hz and resolution of 1,024×800 pixels (1 pixel = 0.6 mm). Fish were startled by tapping the bottom of the tank with a length of PVC pipe (Domenici et al., 2004; Harper and Blake 1990) directly in front of the snout of the fish in line with the long-axis of the body. The initial fish heading pre-stimulus, and therefore stimulus direction were designated as 0°. To minimize variation in stimulus orientation as a factor in response variability, and to quantify variability in responses to a constant stimulus direction, video recordings in which the stimulus was not delivered at this orientation to the fish were excluded from the analysis. Defining the frequency distribution of escape trajectories for a given individual required recording of multiple escape responses while avoiding fatigue or desensitization to the stimulus. Previous work with this species established that 10 escape responses interspersed with 3 minute rests result in no detectable change in performance over time (Gerry et al., 2012). Data were collected in groups of up to 10 responses, with a minimum of 2 hours rest between groups. No more than 2 groups of up to 10 responses were recorded per fish per day. A total of 641 escape responses from the 15 fish were analysed for angular changes during the escape, and of these 272 escape responses from 7 fish further analysed to quantify centre of mass (COM) motion. Data for any given fish were collected within a 4 day period.

Video sequences were downloaded to a personal computer using AOS Digital Imaging software (AOS Technologies, Baden Daettwil, Switzerland). The COM of bluegill sunfish is located approximately 40% of total body length from the snout when the fish is in a straight position (Tytell and Lauder, 2008), although the true COM shifts from the straight body COM location during body bending (Wakeling, 2006), this is typically taken as an indicator of COM position for tracking purposes (Domenici and Blake, 1997). This location on the midline and the snout of each fish were manually tracked using Image J. Position-time data were smoothed using a smoothing spline interpolation in the application Igor Pro (ver. 6.2,Wavemetrics, Lake Oswego, OR). This method is similar to the cubic spline algorithm recommended by Walker (Walker, 1998) for calculating velocities and accelerations from position data. The level of smoothing was dictated by the standard deviation of the raw position data which is used as a smoothing factor in the algorithm. Smoothed COM position data were differentiated to obtain COM velocity, and velocity was differentiated to obtain COM acceleration. The COM and snout position data were used to calculate the heading of the fish. The body axis between the COM and snout is inflexible, and the vector between these two points indicates fish heading. The heading angle of the fish relative to the Y-direction (*θ*) was calculated as:
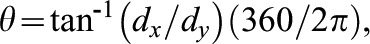
(1)where *d_x_* and *d_y_* are the distances between the COM and snout in the X and Y directions.

Escape responses are typically divided into two kinematic phases (Domenici and Blake, 1997; Wakeling, 2006). Phase 1 consists of the initial C-bend, and phase 2 the subsequent reverse tail stroke. These can be defined on the basis of snout angular velocity. Phase 1 consists of an initial velocity peak, decreasing transiently to 0 at the end of phase 1 before a second angular velocity peak of opposite sign associated with phase 2, again decreasing to 0 at the completion of this phase (Domenici and Blake, 1997; Tytell et al., 2008). A third stage may also be defined in which manoeuvres subsequent to stages 1 and 2 establish the final trajectory of the fish relative to its pre-escape orientation (Weihs, 1973). Variation in behaviour after completion of stage 1 meant that stage 2 could not be consistently defined from body kinematics. For example in the absence of a well defined counter-movement after the initial C-bend, the typical patterns of snout angular velocity change that define phases 1 and 2 may be absent. For the present study we report stage 1 angles, the final escape angle established after completion of any post-stage 1 movements, and the difference between these two angles, termed the steering angle.

### Statistical analyses

If the development of high velocities and accelerations is restricted to a relatively narrow range of body movements by hydrodynamic factors and the axial mechanics of the body, then within a frequency distribution of kinematic variables the most frequently adopted patterns of movement may be associated with high performance. Conversely, infrequently observed behaviours at the margins of the distribution may be associated with low performance. We used regression analyses to determine whether there was a relationship between escape performance (as indicated by peak COM velocity and acceleration, average COM velocity across stages 1 and 2, and COM displacement across stages 1 and 2) and the frequency distributions of stage 1 and escape angles for each individual.

The frequency distributions of the stage 1 and final escape angles were estimated from histograms by applying a standard approach to divide the data for each fish into 1+log_2_(*n*) bins, where *n* was the number of observations (Sturges, 1926). Frequency distributions were normalized to a probability density with an integral of 1 across the observed data range. Fourth order polynomials were fitted to the histograms to provide an estimate of the continuous probability density function for each angle distribution. If escape performance was greatest at the most frequently used escape angles there should be a positive correlation between the performance metrics and the probability density function of the angle distribution. Pearson's product-moment coefficient (PPMC) was used as an indicator of the sign and strength of any performance-probability density relationships. A two tailed t-test was also applied within a linear regression analysis to determine whether the slope of each performance-probability relationship was detectably different from zero.

If modulation of the escape direction produced by steering after completion of stage 1 reduces performance there should be a negative relationship between steering angle magnitude and performance. As steering angles are changes in direction defined relative to fish heading at the end of stage 1 (negative values represent a reversal of turn direction and positive values a continuation of the stage 1 turn direction), and have a relatively narrow range, the data do not have a circular distribution and are suitable for analysis with linear statistical models. ANOVA with a fish identifier as a random factor and turn direction (left vs. right) as a fixed factor was used to test for inter-individual differences in steering angle. PPMC was used to determine the strength and sign of relationships between performance (peak COM velocity and acceleration, average COM velocity, and COM displacement) and steering angle. A two tailed t-test was also applied within a linear regression analysis to determine whether the slope of each performance-probability relationship was detectably different from zero. Data were tested for normality using a Kolmogorov–Smirnov test (p<0.05) and Levene's equality of error variances test (p<0.05). All data were log-transformed to achieve normality. Negative values were adjusted by absolute value transformation before log transformation. Untransformed data are presented in figures.

Stage 1 and escape angle data were analysed for uniformity and left–right symmetry using a circular statistics package (Oriana, ver. 3.21, Kovach Computing Services, Pentraeth,UK). Rayleigh's test established that angle distributions were non-uniform for both left and right turns in all fish (Rayleigh, p<0.05). Circular variance, equivalent to a coefficient of variation for non-directional data, was used as a relative indicator of the dispersion of the distributions, with 0 indicating concentration at a single direction. Frequency distributions for left and right turns for each fish were compared using a Mardia-Watson-Wheeler test (Mardia, 1972). This is a nonparametric test for differences between samples of circularly distributed data. Where no differences in the angle distributions were detected between left and right turns, data were combined for further analysis. Multiple pair wise comparisons based on the Mardia-Watson-Wheeler test were also used to test for inter-individual differences in stage 1 and final escape angle. To account for the use of multiple comparisons the experiment-wise error rate was adjusted using a sequentially rejective multiple test procedure applying Ryan's Q (Ryan, 1960).

The behavioural repeatability, *r*, was calculated as follows:
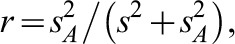
(2)where 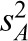 is the is the among-groups variance component and *s*^2^ is the within-group variance (Lessells and Boag, 1987; Nakagawa and Schielzeth, 2010). In this context *s*^2^ is the variance in behaviour exhibited by individuals, and 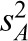 is the variance in behaviour between individuals. *r* therefore indicates the proportion of the total phenotypic variance that is attributable to the between-individual variance. The variance components were calculated from the mean squares derived from one-way ANOVA with an identifier for each individual as a random factor as follows:
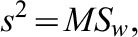
(3)and
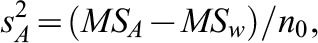
(4)where *MS_A_* and *MS_W_* are the across and within groups mean squares and *n*_0_ is related to the number of observations obtained per individual as follows:
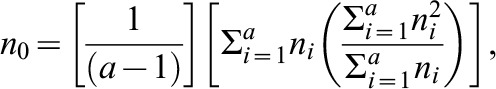
(5)where *a* is the number of individuals and *n*_i_ is the sample size of the *i*th group. ANOVA is generally not suitable for analysis of directional data as the frequency distribution may be ‘wrapped’ around a circle due to the equivalency of 0° and 360°. However, given that the starting angle was defined as 0° and that no angular changes exceeded 360°, mean squares values obtained by ANOVA give a reasonable estimate of repeatability in this case. The F-statistic and p-value obtained by ANOVA indicate whether the repeatability is significantly greater than zero (Donner, 1986). To account for the use of multiple comparisons the experiment-wise error rate was adjusted using a sequentially rejective multiple test procedure applying Ryan's Q (Ryan, 1960). Linear statistical analyses were carried out using the application PASW Statistics (Version 18, SPSS, Chicago, IL, USA).

## References

[b1] Aerts P. (1998). Vertical jumping in Galago senegalensis: the quest for an obligate mechanical power amplifier. Philos. Trans. R. Soc. B 353, 1607–1620. 10.1098/rstb.1998.0313

[b2] Arnott S. A. Neil D. M. Ansell A. D. (1999). Escape trajectories of the brown shrimp crangon crangon, and a theoretical consideration of initial escape angles from predators. J. Exp. Biol. 202, 193–209. 9851908 10.1242/jeb.202.2.193

[b3] Askew G. N. Marsh R. L. (2002). Muscle designed for maximum short-term power output: quail flight muscle. J. Exp. Biol. 205, 2153–2160. 12110648 10.1242/jeb.205.15.2153

[b4] Bell A. M. Hankison S. J. Laskowski K. L. (2009). The repeatability of behaviour: a meta-analysis. Anim. Behav. 77, 771–783. 10.1016/j.anbehav.2008.12.022 24707058 PMC3972767

[b5] Bonine K. E. Garland T. G. Jr (1999). Sprint performance of phrysonomatid lizards, measured on a high-speed treadmill, correlates with hindlimb length. J. Zool. (Lond.) 248, 255–265. 10.1111/j.1469-7998.1999.tb01201.x

[b7] Catania K. C. (2009). Tentacled snakes turn C-starts to their advantage and predict future prey behavior. Proc. Natl. Acad. Sci. USA 106, 11183–11187. 10.1073/pnas.0905183106 19549832 PMC2699377

[b8] Cohen J. (1988). Statistical Power Analysis for the Behavioral Sciences, 2nd edn Hillsdale, NJ: Erlbaum.

[b9] Domenici P. (2002). The visually mediated escape response in fish: predicting prey responsiveness and the locomotor behaviour of predators and prey. Mar. Freshwat. Behav. Physiol. 35, 87–110. 10.1080/10236240290025635

[b10] Domenici P. (2010a). Context-dependent variability in the components of fish escape response: integrating locomotor performance and behavior. J. Exp. Zool. 313A, 59–79. 10.1002/jez.580 20073047

[b11] Domenici P. (2010b). Escape responses in fish: kinematics, performance and behaviour. Fish Locomotion: An Eco-Ethological Perspective Domenici P Kapoor B G, ed123–170.Boca Raton, FL: CRC Press.

[b12] Domenici P. Batty R. S. (1997). Escape behaviour of solitary herring (Clupea harengus) and comparisons with schooling individuals. Mar. Biol. 128, 29–38. 10.1007/s002270050065

[b13] Domenici P. Blake R. W. (1991). The kinematics and performance of the escape response of the escape response in the angelfish (Pterophyllum eimekei). J. Exp. Biol. 156, 187–205.

[b14] Domenici P. Blake R. W. (1993). Escape trajectories in angelfish (Pterophyllum eimekei). J. Exp. Biol. 177, 253–272.

[b15] Domenici P. Blake R. (1997). The kinematics and performance of fish fast-start swimming. J. Exp. Biol. 200, 1165–1178. 9319004 10.1242/jeb.200.8.1165

[b16] Domenici P. Standen E. M. Levine R. P. (2004). Escape manoeuvres in the spiny dogfish (Squalus acanthias). J. Exp. Biol. 207, 2339–2349. 10.1242/jeb.01015 15159438

[b17] Domenici P. Booth D. Blagburn J. M. Bacon J. P. (2008). Cockroaches keep predators guessing by using preferred escape trajectories. Curr. Biol. 18, 1792–1796. 10.1016/j.cub.2008.09.062 19013065 PMC2678410

[b18] Domenici P. Blagburn J. M. Bacon J. P. (2011a). Animal escapology I: theoretical issues and emerging trends in escape trajectories. J. Exp. Biol. 214, 2463–2473. 10.1242/jeb.029652 21753039 PMC4495464

[b19] Domenici P. Blagburn J. M. Bacon J. P. (2011b). Animal escapology II: escape trajectory case studies. J. Exp. Biol. 214, 2474–2494. 10.1242/jeb.053801 21753040 PMC3135389

[b20] Donner A. (1986). A review of inference procedures for the intraclass correlation coefficient in the one-way random effects model. Int. Stat. Rev. 54, 67–82. 10.2307/1403259

[b21] Eaton R. C. Emberley D. S. (1991). How stimulus direction determines the trajectory of the Mauthner-initiated escape response in a teleost fish. J. Exp. Biol. 161, 469–487. 1757775 10.1242/jeb.161.1.469

[b22] Eaton R. C. DiDomenico R. Nissanov J. (1988). Flexible body dynamics of the goldfish C-start: implications for reticulospinal command mechanisms. J. Neurosci. 8, 2758–2768. 3411353 10.1523/JNEUROSCI.08-08-02758.1988 PMC6569417

[b23] Eaton R. C. Lee R. K. K. Foreman M. B. (2001). The Mauthner cell and other identified neurons of the brainstem escape network of fish. Prog. Neurobiol. 63, 467–485. 10.1016/S0301-0082(00)00047-2 11163687

[b24] Foreman M. B. Eaton R. C. (1993). The direction change concept for reticulospinal control of goldfish escape. J. Neurosci. 13, 4101–4113. 8410180 10.1523/JNEUROSCI.13-10-04101.1993 PMC6576370

[b25] Fuiman L. A. Cowan J. H. (2003). Behavior and recruitment success in fish larvae: repeatability and covariation of survival skills. Ecology 84, 53–67. 10.1890/0012-9658(2003)084\[0053:BARSIF\]2.0.CO;2

[b26] Gahtan E. Sankrithi N. Campos J. B. O'Malley D. M. (2002). Evidence for a widespread brain stem escape network in larval zebrafish. J. Neurophysiol. 87, 608–614. 11784774 10.1152/jn.00596.2001

[b27] Garland T. Jr (1985). Ontogenetic and individual variation in size, shape, and speed in the Australian agamid lizard Amphibolurus nuchalis. J. Zool. 207, 425–439. 10.1111/j.1469-7998.1985.tb04941.x

[b28] Gerry S. P. Wang J. Ellerby D. J. (2011). A new approach to quantifying morphological variation in bluegill Lepomis macrochirus. J. Fish Biol. 78, 1023–1034. 10.1111/j.1095-8649.2011.02911.x 21463305

[b29] Gerry S. P. Robbins A. Ellerby D. J. (2012). Variation in fast-start performance within a population of polyphenic bluegill (Lepomis macrochirus). Physiol. Biochem. Zool. 85, 694–703. 10.1086/667593 23099466

[b30] Gibson S. Johnston I. A. (1995). Scaling relationships, individual variation and the influence of temperature on maximum swimming speed in early settled stages of the turbot Scophthalmus maximus. Mar. Biol. 121, 401–408. 10.1007/BF00349449

[b31] Gleeson T. T. Harrison J. M. (1988). Muscle composition and its relation to sprint running in the lizard Dipsosaurus dorsalis. Am. J. Physiol. 255, R470–R477. 3414842 10.1152/ajpregu.1988.255.3.R470

[b32] Hall S. J. Wardle C. S. MacLennan D. N. (1986). Predator evasion in a fish school-test of a model for the fountain effect. Mar. Biol. 91, 143–148. 10.1007/BF00397579

[b33] Harper D. G. Blake R. W. (1990). Fast-start performance of rainbow trout Salmo gairdneri and northern pike Esox lucius. J. Exp. Biol. 150, 321–342.

[b34] Henry H. T. Ellerby D. J. Marsh R. L. (2005). Performance of guinea fowl Numida meleagris during jumping requires storage and release of elastic energy. J. Exp. Biol. 208, 3293–3302. 10.1242/jeb.01764 16109891

[b35] Humphries D. A. Driver P. M. (1970). Protean defence by prey animals. Oecologia 5, 285–302. 10.1007/BF00815496 28309783

[b36] Husak J. F. (2006). Does survival depend on how fast you can run or how fast you do run? Funct. Ecol. 20, 1080–1086. 10.1111/j.1365-2435.2006.01195.x

[b37] Jabłoński P. G. Strausfeld N. J. (2001). Exploitation of an ancient escape circuit by an avian predator: relationships between taxon-specific prey escape circuits and the sensitivity to visual cues from the predator. Brain Behav. Evol. 58, 218–240. 10.1159/000057565 11964498

[b38] Kasapi M. A. Domenici P. Blake R. W. Harper D. (1993). The kinematics and performance of escape responses of the knifefish Xenomystus nigri. Can. J. Zool. 71, 189–195. 10.1139/z93-026

[b39] Korn H. Faber D. S. (2005). The Mauthner cell half a century later: a neurobiological model for decision-making? Neuron 47, 13–28. 10.1016/j.neuron.2005.05.019 15996545

[b40] Langerhans R. B. Layman C. A. Shokrollahi A. M. DeWitt T. J. (2004). Predator-driven phenotypic diversification in Gambusia affinis. Evolution 58, 2305–2318. 10.1111/j.0014-3820.2004.tb01605.x 15562692

[b41] Lessells C. M. Boag P. T. (1987). Unrepeatable repeatabilities: a common mistake. Auk 104, 116–121. 10.2307/4087240

[b42] Lind J. Cresswell W. (2005). Determining the fitness consequences of antipredation behaviour. Behav. Ecol. 16, 945–956. 10.1093/beheco/ari075

[b43] Mardia K. V. (1972). A multi-sample uniform scores test on a circle and its parametric competitor. J. R. Stat. Soc. B 34, 102–113.

[b44] Marras S. Killen S. S. Claireaux G. Domenici P. McKenzie D. J. (2011). Behavioural and kinematic components of the fast-start escape response in fish: individual variation and temporal repeatability. J. Exp. Biol. 214, 3102–3110. 10.1242/jeb.056648 21865523

[b45] Meager J. J. Domenici P. Shingles A. Utne-Palm A. C. (2006). Escape responses in juvenile Atlantic cod Gadus morhua L.: the effects of turbidity and predator speed. J. Exp. Biol. 209, 4174–4184. 10.1242/jeb.02489 17023610

[b46] Metcalfe W. K. Mendelson B. Kimmel C. B. (1986). Segmental homologies among reticulospinal neurons in the hindbrain of the zebrafish larva. J. Comp. Neurol. 251, 147–159. 10.1002/cne.902510202 3782495

[b47] Miles D. B. (2004). The race goes to the swift: fitness consequences of variation in sprint performance in juvenile lizards. Evol. Ecol. Res. 6, 63–75.

[b48] Nakagawa S. Schielzeth H. (2010). Repeatability for Gaussian and non-Gaussian data: a practical guide for biologists. Biol. Rev. Camb. Philos. Soc. 85, 935–956. 10.1111/j.1469-185X.2010.00141.x 20569253

[b49] Nauen J. C. Shadwick R. E. (2001). The dynamics and scaling of force production during the tail-flip escape response of the California spiny lobster Panulirus interruptus. J. Exp. Biol. 204, 1817–1830. 11316502 10.1242/jeb.204.10.1817

[b50] Nissanov J. Eaton R. C. DiDomenico R. (1990). The motor output of the Mauthner cell, a reticulospinal command neuron. Brain Res. 517, 88–98. 10.1016/0006-8993(90)91012-6 2376010

[b51] Nowroozi B. N. Brainerd E. L. (2013). X-ray motion analysis of the vertebral column during the startle response in striped bass, Morone saxatilis. J. Exp. Biol. 216, 2833–2842. 10.1242/jeb.085118 23842627

[b52] O'Malley D. M. Kao Y.-H. Fetcho J. R. (1996). Imaging the functional organization of zebrafish hindbrain segments during escape behaviors. Neuron 17, 1145–1155. 10.1016/S0896-6273(00)80246-9 8982162

[b53] Roberts T. J. Marsh R. L. (2003). Probing the limits to muscle-powered accelerations: lessons from jumping bullfrogs. J. Exp. Biol. 206, 2567–2580. 10.1242/jeb.00452 12819264

[b54] Ryan T. A. (1960). Significance tests for multiple comparison of proportions, variances, and other statistics. Psychol. Bull. 57, 318–328. 10.1037/h0044320 14440422

[b55] Sturges H. A. (1926). The choice of a class interval. J. Am. Stat. Assoc. 21, 65–66. 10.1080/01621459.1926.10502161

[b56] Sutton G. P. Burrows M. (2011). Biomechanics of jumping in the flea. J. Exp. Biol. 214, 836–847. 10.1242/jeb.052399 21307071

[b57] Tytell E. D. Lauder G. V. (2008). Hydrodynamics of the escape response in bluegill sunfish, Lepomis macrochirus. J. Exp. Biol. 211, 3359–3369. 10.1242/jeb.020917 18931309 PMC2669901

[b58] Tytell E. D. Standen E. M. Lauder G. V. (2008). Escaping Flatland: three-dimensional kinematics and hydrodynamics of median fins in fishes. J. Exp. Biol. 211, 187–195. 10.1242/jeb.008128 18165246

[b59] Wainwright P. C. Mehta R. S. Higham T. E. (2008). Stereotypy, flexibility and coordination: key concepts in behavioral functional morphology. J. Exp. Biol. 211, 3523–3528. 10.1242/jeb.007187 18978215

[b60] Wakeling J. M. (2006). Fast-start mechanics. Fish Biomechanics: Fish Physiology, Vol. 23 Shadwick R E Lauder G V, ed333–368.San Diego, CA: Academic Press.

[b61] Wakeling J. M. Johnston I. A. (1999). Body bending during fast-starts in fish can be explained in terms of muscle torque and hydrodynamic resistance. J. Exp. Biol. 202, 675–682. 10021321 10.1242/jeb.202.6.675

[b62] Walker J. A. (1998). Estimating velocities and accelerations of animal locomotion: a simulation experiment comparing numerical differentiation algorithms. J. Exp. Biol. 201, 981–995.

[b63] Walker J. A. Ghalambor C. K. Griset O. L. McKenney D. Reznick D. N. (2005). Do faster starts increase the probability of evading predators? Funct. Ecol. 19, 808–815. 10.1111/j.1365-2435.2005.01033.x

[b64] Watkins T. B. (1996). Predator-mediated selection on burst swimming performance in tadpoles of the Pacific tree frog, Pseudacris regilla. Physiol. Zool. 69, 154–167.

[b65] Webb P. W. (1978). Fast-start performance and body form in seven species of teleost fish. J. Exp. Biol. 74, 211–226.

[b66] Weihs D. (1973). The mechanism of rapid starting of slender fish. Biorheology 10, 343–350. 4772008 10.3233/bir-1973-10308

[b67] Weihs D. Webb P. W. (1984). Optimal avoidance and evasion tactics in predator–prey interactions. J. Theor. Biol. 106, 189–206. 10.1016/0022-5193(84)90019-5

[b68] Weiss S. A. Zottoli S. J. Do S. C. Faber D. S. Preuss T. (2006). Correlation of C-start behaviors with neural activity recorded from the hindbrain in free-swimming goldfish (Carassius auratus). J. Exp. Biol. 209, 4788–4801. 10.1242/jeb.02582 17114411

[b69] Westneat M. W. Hale M. E. McHenry M. J. Long J. H. (1998). Mechanics of the fast-start: muscle function and the role of intramuscular pressure in the escape behavior of amia calva and polypterus palmas. J. Exp. Biol. 201, 3041–3055. 9787124 10.1242/jeb.201.22.3041

[b70] Wöhl S. Schuster S. (2007). The predictive start of hunting archer fish: a flexible and precise motor pattern performed with the kinematics of an escape C-start. J. Exp. Biol. 210, 311–324. 10.1242/jeb.02646 17210967

[b71] Zani P. A. Jones T. D. Neuhaus R. A. Milgrom J. E. (2009). Effect of refuge distance on escape behaviour of side-blotched lizards (Uta stansburiana). Can. J. Zool. 87, 407–414. 10.1139/Z09-029

